# Anti-Hyperlipidemic Effect of Fucoidan Fractions Prepared from Iceland Brown Algae *Ascophyllum nodosum* in an Hyperlipidemic Mice Model

**DOI:** 10.3390/md21090468

**Published:** 2023-08-26

**Authors:** Yunhai He, Yutong Li, Peili Shen, Shangkun Li, Linsong Zhang, Qiukuan Wang, Dandan Ren, Shu Liu, Demeng Zhang, Hui Zhou

**Affiliations:** 1 College of Food Science and Engineering, Dalian Ocean University, Dalian 116023, China; 2 State Key Laboratory of Marine Food Processing & Safety Control, Qingdao Bright Moon Seaweed Group Co., Ltd., Qingdao 266400, China; 3 Key Laboratory of Aquatic Product Processing and Utilization of Liaoning Province, Dalian Ocean University, Dalian 116023, China; 4 National R&D Branch Center for Seaweed Processing, Dalian Ocean University, Dalian 116023, China

**Keywords:** *Ascophyllum nodosum*, fucoidan, anti-hyperlipidemic, antioxidant enzyme, lipoprotein metabolism, gut flora

## Abstract

*Ascophyllum nodosum*, a brown algae abundantly found along the North Atlantic coast, is recognized for its high polysaccharide content. In this study, we investigated the anti-hyperlipidemic effect of fucoidans derived from *A. nodosum*, aiming to provide information for their potential application in anti-hyperlipidemic therapies and to explore comprehensive utilization of this Iceland brown seaweed. The crude fucoidan prepared from *A. nodosum* was separated using a diethylethanolamine column, resulting in two fucoidan fractions, AFC-1 and AFC-2. Both fractions were predominantly composed of fucose and xylose. AFC-1 exhibited a higher sulfate content of 27.8% compared to AFC-2 with 17.0%. AFC-2 was primarily sulfated at the hydroxy group of C2, whereas AFC-1 was sulfated at both the hydroxy groups of C2 and C4. To evaluate the anti-hyperlipidemic effect, a hyperlipidemia mouse model was established by feeding mice a high-fat diet. The effects of AFC-1, AFC-2, and the crude extract were investigated, with the drug atorvastatin used as a positive comparison. Among the different fucoidan fractions and doses, the high dose of AFC-2 administration demonstrated the most significant anti-hyperlipidemic effect across various aspects, including physiological parameters, blood glucose levels, lipid profile, histological analysis, and the activities of oxidative stress-related enzymes and lipoprotein-metabolism-related enzymes (*p* < 0.05 for the final body weight and *p* < 0.01 for the rest indicators, compared with the model group), and its effect is comparable to the atorvastatin administration. Furthermore, fucoidan administration resulted in a lower degree of loss in gut flora diversity compared to atorvastatin administration. These findings highlight the significant biomedical potential of fucoidans derived from *A. nodosum* as a promising therapeutic solution for hypolipidemia.

## 1. Introduction

*Ascophyllum nodosum* is a marine brown algae that thrives along the North Atlantic coast [1]. Apart from its ecological significance, this algae has garnered substantial attention for its industrial applications in alginate extraction, as well as its utilization in agriculture for fertilizer and feed production. In the food industry, it is commonly processed into seaweed powder, which finds its way into various culinary and functional food products. Notably, *Ascophyllum nodosum* contains fucoidan, a typical brown algal sulfated glycan, which has been the subject of extensive scientific exploration.

Fucoidan is a water-soluble heteropolysaccharide rich in sulfate groups, and has emerged as a fascinating compound due to its diverse biological activities and potential health benefits. It has shown promising properties in various fields, including anti-tumor [2,3,4], anti-viral [5,6], hepatoprotective [7,8], anticoagulant [9,10], anti-inflammatory [11], immunomodulating [12], and hypolipidemic effects [13]. Fucoidan’s unique negatively charged property contributes to its bioactivity and therapeutic potential. Moreover, its natural origin and biocompatibility make it an attractive candidate for applications in the food and biomedicine sectors, with compelling prospects for future development.

As modern society develops, hyperlipidemia has become a major public health concern. Characterized by elevated levels of lipids in the bloodstream, hyperlipidemia poses a significant risk for cardiovascular diseases, including atherosclerosis and coronary heart disease. The alarming rise in cardiovascular diseases has surpassed cancer mortality rates, making it a critical concern [14]. In China alone, over 300 million individuals are affected, with a staggering statistic of one patient dying every 9 s [15]. The effective management of blood lipids plays a pivotal role in disease prevention and treatment. Of particular significance are total cholesterol (TC) and LDL cholesterol (LDL-C), which exhibit a strong correlation with the incidence of coronary heart disease [16]. While chemical drugs are currently the primary treatment for hyperlipidemia, their side effects and potential harm to the liver and kidneys have led to a growing interest in finding natural and harmless therapeutic agents, making it a prominent area of research [17].

In recent years, gut flora has been found to be a key environmental factor regulating body metabolism. Research shows that it is involved in the occurrence and development of obesity, diabetes, atherosclerosis, and other chronic diseases, and plays a vital role in regulating host lipid metabolism [18]. Hyperlipidemia leads to an imbalance in gut flora, and modifying gut flora may be a new method to treat hyperlipidemia [19].

Based on the above-mentioned background, this study aimed to explore the anti-hyperlipidemic effect of fucoidan fractions prepared from *Ascophyllum nodosum* sourced from Iceland. Two fucoidan fractions were derived using an anion-exchange method, and their compositional information was further investigated. Using a hyperlipidemia mouse model, the effects of the fucoidan crude extract and fractions on physiological parameters, lipid profiles, and relevant enzymatic activities were investigated. Histological studies of liver tissues and gut flora compositional analysis were also conducted to provide further information. While some recent studies have explored the anti-hyperlipidemic activity of fucoidan prepared from common brown seaweed species and reported encouraging results [20,21,22,23,24], no research has been reported using fucoidan prepared from *Ascophyllum nodosum*. This study aims to provide insights into the biomedical applications of fucoidans and their potential as a natural anti-hyperlipidemic product, as well as information for the future comprehensive utilization of *Ascophyllum nodosum* for high-value product production.

## 2. Results

### 2.1. Separation and Compositional Analysis of A. nodosum Fucoidans (AN-FUC)

**Figure 1:**
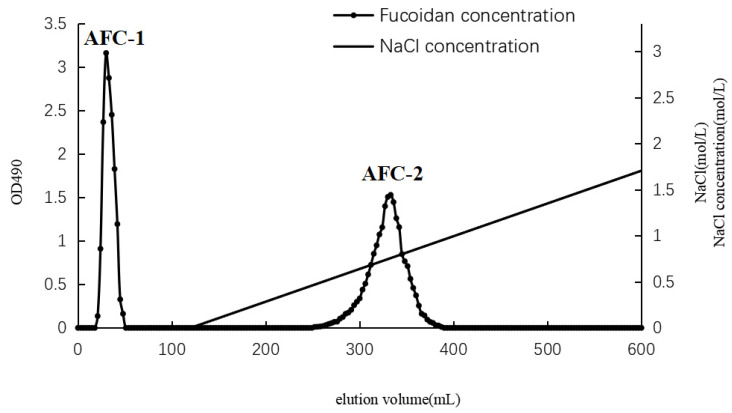
Chromatography of fucoidan separation using DEAE column.

The separation of AN-FUC using the Diethylaminoethyl (DEAE)-Sepharose Fast Flow anion exchange column is illustrated in Figure 1. Two distinct fractions, AFC-1 and AFC-2, were obtained. AFC-1 was eluted with water, while AFC-2 was eluted with an increasing salinity of the mobile phase. The symmetrical peaks observed in the chromatography indicate a relatively high purity of the two fractions. The carbohydrate content, sulfate content, and monosaccharide composition of each fucoidan fraction are summarized in Table 1. AFC-2 exhibited the highest carbohydrate content, while AFC-1 had the highest sulfate content. The dominant monosaccharides in both fractions were fucose and xylose, with small amounts of mannose, glucose, rhamnose, galactose, and glucuronic acid also detected.

### 2.2. Structural Features of Fucoidans from A. nodosum

**Figure 2:**
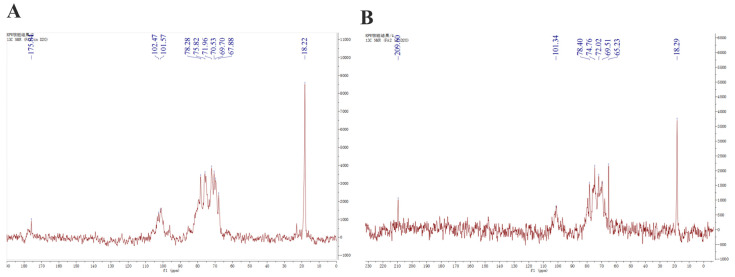
^13^C NMR spectra of fucoidans from A. nodosum. (**A**): AFC-1, (**B**): AFC-2.

**Figure 3:**
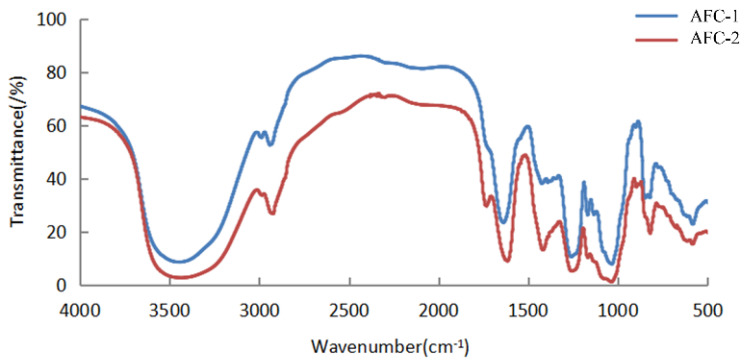
The FT-IR spectra of fucoidans from A. nodosum.

The ^13^C NMR spectra of fucoidans from A. nodosum, as shown in Figure 2, confirmed the presence of a high fucose content in AFC-1 and AFC-2, indicated by the significant fucose C6 signal peaks at 18.22 ppm and 18.29 ppm, respectively. The major anomeric C1 signal at 101–102 ppm was assigned to α-L-fucose. Both AFC-1 and AFC-2 exhibited complex signals in the range of 69–85 ppm, suggesting heterogeneous substitution of the hydroxy group in the sugar with either glycosidic linkage or sulfate group. The FT-IR spectra, depicted in Figure 3, displayed typical glycan features of AFC-1 and AFC-2. The broad bands observed at 3600–3200 cm^−1^ were assigned to the deformation of O-H. The bands around 2939 cm^−1^ and 2923 cm^−1^ corresponded to C-H stretching vibrations. AFC-1 and AFC-2 exhibited absorption bands at around 1035 cm^−1^ and 1260 cm^−1^, respectively, which were attributed to C-O and asymmetric O-S-O stretching vibrations, respectively [1]. Both AFC-1 and AFC-2 displayed absorption peaks at 821 cm^−1^ and 819 cm^−1^, indicating equatorial C-O-S stretching vibrations caused by sulfation of the hydroxy group at C2. Additionally, AFC-1 exhibited an absorption peak around 847 cm^−1^, suggesting the presence of an axial C-O-S stretching vibration, corresponding to the hydroxy group at C4 [2].

### 2.3. Effect of Fucoidan Administration on Physiological Parameters and Blood Glucose Level

The impact of fucoidan administration on physiological parameters and blood glucose levels is summarized in Table 2. The initial body weight did not show significant differences among the groups. However, the model group (no drug administered, high-fat diet fed) exhibited a significant difference in body weight compared to the control group (*p* < 0.05) at the end point, indicating the successful establishment of the model. The drug group (atorvastatin administered, high-fat diet fed) displayed a significant difference in body weight compared to the model group (*p* < 0.01) and showed similar body weight to the control group, suggesting a notable antihyperlipidemic effect of the drug. Similar patterns were observed for the liver index and blood glucose levels, with the model group showing significantly higher values compared to the control group, while the drug group demonstrated higher values than the control group but lower than the model group, with significant differences. A normal distribution test was performed before statistical analysis using one-way analysis of variance (ANOVA) (*p* < 0.05, the same below).

The different fucoidan groups exhibited varied effects on these parameters, with the medium and high dose groups of AFC-2 demonstrating superior effects compared to other groups. These groups showed significant differences when compared to the model group, indicating notable effects on all three parameters.

### 2.4. Effect of Fucoidan Administration on Lipid Profile

**Figure 4:**
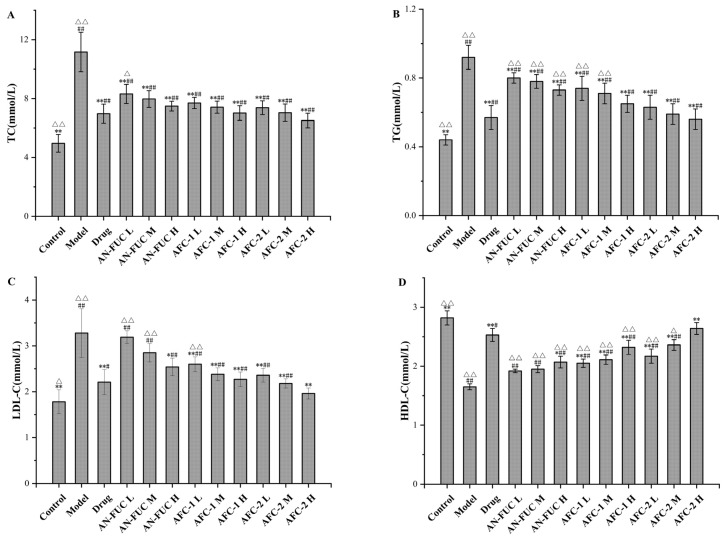
Effects of fucoidan on lipid profile after 28 days. (**A**): TC level. (**B**): TG level. (**C**): HDL-C level. (**D**): LDL-C level. Values are expressed as the means ± standard deviation (*n* = 8). **: *p* < 0.01 compared with the model group; *: *p* < 0.05 compared with the model group; ##: *p* < 0.01 compared with the control group; #: *p* < 0.05 compared with the control group; △△: *p* < 0.01 compared with the drug group, △: *p* < 0.05 compared with the drug group.

The differences in lipid profile among the groups are shown in Figure 4. As expected, the model group displayed typical features of hyperlipidemia, with higher levels of total cholesterol (TC), triglycerides (TGs), and low-density lipoprotein cholesterol (LDL-C), but lower levels of high-density lipoprotein cholesterol (HDL-C) compared to the control group. The drug group exhibited significant improvement in the lipid panel compared to the model group but still showed a statistically significant difference from the control group. Most of the fucoidan-administered groups showed improvements in the lipid panel compared to the model group, although to a lesser degree than the drug group. Generally, each fucoidan fraction demonstrated a dose-dependent improvement among the low, medium, and high-dose groups. AFC-1 and AFC-2 showed better effects than the crude extract counterpart at the same dose. Based on the data, the high-dose AFC-2 group demonstrated the most favorable outcomes among the fucoidan groups, judging from the lipid panel data.

### 2.5. Effect of Fucoidan Administration on Oxidative-Stress-Relevant Enzyme Activities

**Figure 5:**
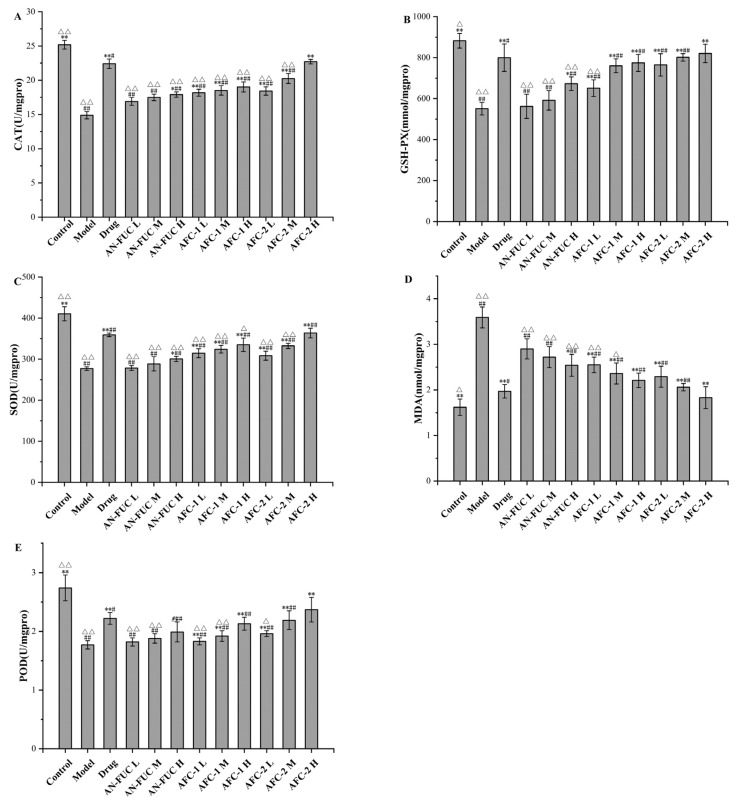
Effects of fucoidan on CAT, GSH-PX, SOD, MDA and POD levels of mice liver tissue. (**A**): CAT; (**B**): GSH-PX; (**C**): SOD; (**D**): MDA; (**E**): POD. Values are expressed as the means ± standard deviation (*n* = 8). **: *p* < 0.01 compared with the model group; *: *p* < 0.05 compared with the model group; ##: *p* < 0.01 compared with the control group; #: *p* < 0.05 compared with the control group; △△: *p* < 0.01 compared with the drug group, △: *p* < 0.05 compared with the drug group.

The levels of oxidative stress-relevant enzymes and markers were determined for each group, as shown in Figure 5. The model group exhibited lower levels of catalase (CAT), glutathione peroxidase (GSH-PX), superoxide dismutase (SOD), and peroxidase (POD) compared to the control group, indicating a compromised antioxidative capacity in the liver caused by hyperlipidemia. Additionally, the model group showed a significant increase in malondialdehyde (MDA) levels, which is a final product of polyunsaturated fatty acid peroxidation, indicating significant oxidative stress in the liver of hyperlipidemic mice.

The drug group demonstrated apparent improvement compared to the model group in all oxidative stress indicators mentioned above, although enzyme function and MDA levels did not fully restore to the normal levels observed in the control group. The fucoidan-administered groups showed a dose-dependent improvement compared to the model group for each fucoidan fraction. Notably, the high dose of AFC-2 exhibited comparable ameliorative effects as atorvastatin on hyperlipidemia-related oxidative stress.

### 2.6. Effect of Fucoidan Administration on Lipoprotein-Metabolism-Related Enzyme Activities

**Figure 6:**
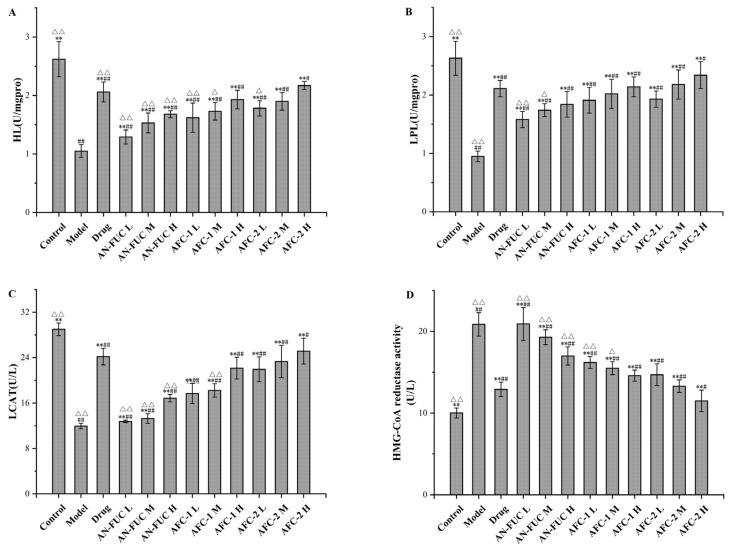
Effects of fucoidan on HL, LPL, LCAT and HMG-CoA reductase activity levels of mice live tissue. (**A**): HL; (**B**): LPL; (**C**): LCAT; (**D**): HMG-CoA reductase activity. Values are expressed as the means ± standard deviation (*n* = 8). **: *p* < 0.01 compared with the model group; ##: *p* < 0.01 compared with the control group; #: *p* < 0.05 compared with the control group; △△: *p* < 0.01 compared with the drug group, △: *p* < 0.05 compared with the drug group.

The effects of fucoidans on lipoprotein-metabolism-related enzyme activities are shown in Figure 6. Compared to the control group, the model group exhibited a 51.99% increase in hepatic 3-hydroxy-3-methylglutaryl coenzyme A (HMG-CoA) reductase activity, along with significant reductions of 59.92%, 63.88%, and 58.81% in hepatic liver lipase (HL), lipoprotein lipase (LPL), and lecithin-cholesterolacyltransferase (LCAT) activities, respectively, compared to the control group. By administering atorvastatin and varying concentrations of fucoidan sulfate, the levels of HMG-CoA in the liver decreased, while the levels of HL, LPL, and LCAT increased compared to the model group, indicating the pharmaceutical effects of fucoidan on hyperlipidemia in a dose-dependent manner. Once again, the high-dose AFC-2 group exhibited the most effective outcome among the fucoidan groups.

### 2.7. Histological Observation of Liver Tissues

**Figure 7:**
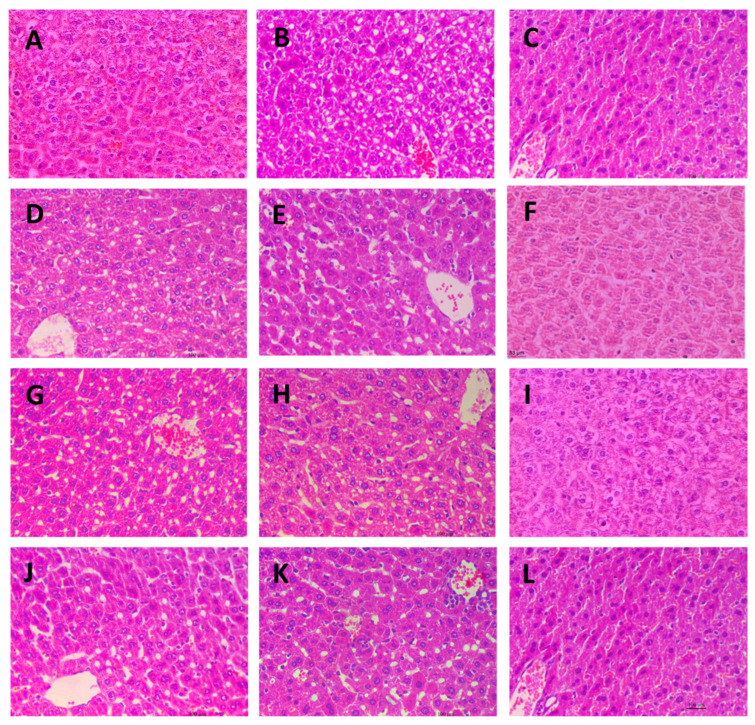
Microscopic images of liver tissues stained with Hematoxylin-Eosin (400×). (**A**): Control group; (**B**): Model group; (**C**): Drug group; (**D**): AN-FUC-L group; (**E**): AN-FUC-M group; (**F**): AN-FUC-H group; (**G**): AFC-1-L group; (**H**): AFC-1-M group; (**I**): AFC-1-H group; (**J**): AFC-2-L group; (**K**): AFC-2-M group; (**L**): AFC-2-H group.

Microscopic images of the livers from different experimental groups are presented in Figure 7. The liver tissue from the control group (Figure 7A), drug group (Figure 7C), and the three high-dose fucoidan groups (Figure 7F,I,L) exhibited a uniform and compact appearance, with closely arranged cells. In contrast, the liver tissue from the model group displayed significant cavitations, likely attributed to the loss of lipid droplets in the paraffin sections. Notably, all the low and medium dose fucoidan-administered groups showed similar microscopic appearances to the model group. These distinct observations provide histological evidence that both the drug and high-dose fucoidan administrations effectively prevent lipid accumulation in the liver.

### 2.8. Effect of Fucoidan Administration on Gut Flora Composition

**Figure 8:**
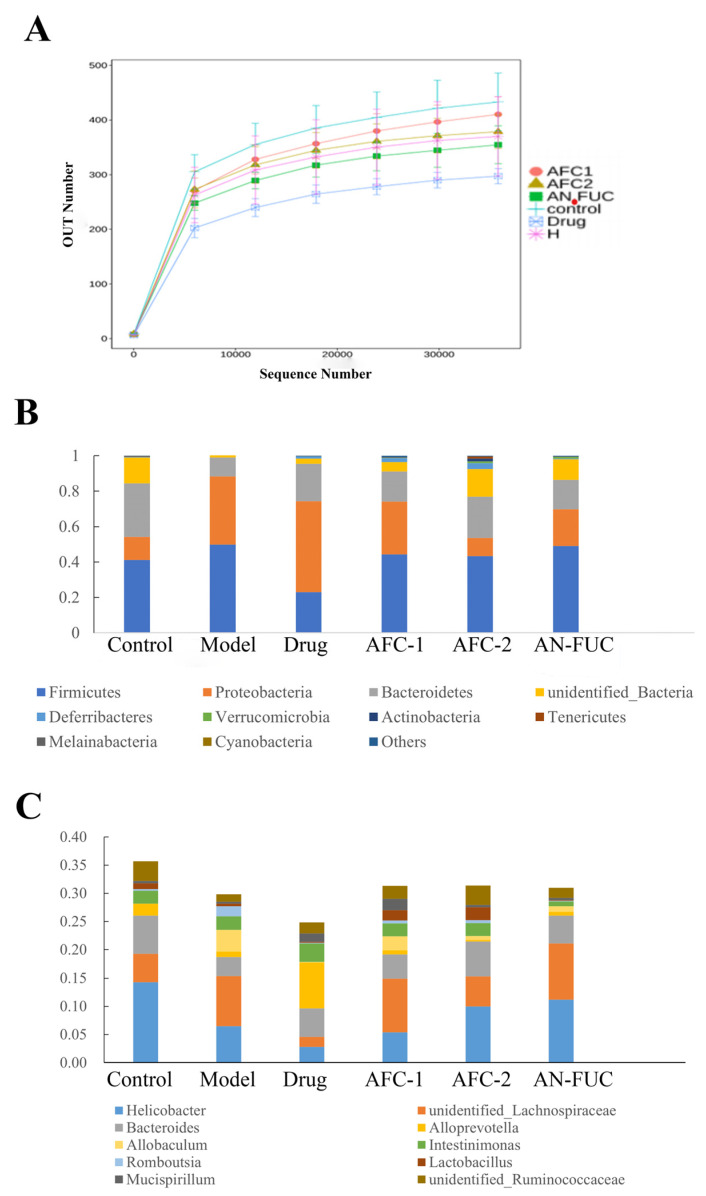
Dilution curve (**A**) of the high-throughput sequencing of the gut flora and relative abundance at phylum level (**B**) and genus level (**C**).

The dilution curves are commonly used to depict the diversity of samples within a group, providing insights into the sequencing data volume and species richness. When the curve flattens out, it indicates that increasing sequencing data does not significantly contribute to obtaining new operational taxonomic units (OTUs), thereby reflecting the saturation of the sequencing depth. Figure 8A illustrates that the number of OTUs rapidly increased and then plateaued with increasing sequencing depth, indicating that the sequencing volume in this experiment was sufficient to capture most of the microorganisms and reflect the basic composition of the intestinal flora in each group. Moreover, Figure 8A shows that the high-fat diet resulted in a reduction in the diversity of the mouse intestinal flora. Notably, the atorvastatin group exhibited the lowest diversity, suggesting that the drug had a substantial impact on the diversity of the intestinal flora.

Figure 8B,C demonstrate the proportional changes in gut flora for different groups. At the phylum level, the majority of the groups fed with a high-fat diet exhibited increased proportions of Proteobacteria and reduced proportions of Bacteroidetes. Additionally, the atorvastatin-administered mice showed a notable decrease in the proportion of Firmicutes. At the genus level, all the groups fed with a high-fat diet exhibited decreased proportions of Helicobacter. The drug group showed an exceptional increase in Alloprevotella, while the other high-fat diet groups showed an increased proportion of an unidentified Lachnospiraceae genus.

## 3. Discussion

This study explored the anti-hyperlipidemic effects of fucoidan components derived from Icelandic brown algae *Ascophyllum nodosum* in a mouse model. We examined physiological parameters, lipid profiles, and paraffin sections of liver tissues to confirm the anti-hyperlipidemic effects of fucoidan administration. Further investigation involved assessing the activity of oxidative stress-related enzymes to understand the mechanisms behind fucoidan’s anti-hyperlipidemic activity. The experimental results consistently demonstrate that fucoidan administration prevented significant reductions in the activities of oxidative stress-related enzymes and decreased levels of the oxidative stress indicator MDA, which had been notably increased in the model group.

Additionally, we examined the activity of lipoprotein-metabolism-related enzymes and found that fucoidan administration prevented significant changes in enzyme activities observed in the model group. Specifically, it alleviated decreases in HL, LPL, and LCAT activities while also inhibiting the increase in HMG-CoA reductase activity. HL, LPL, and LCAT are all crucial enzymes involved in lipid metabolism. The lower levels of these enzymes in the model group indicated impaired lipid metabolism, which was improved by the administration of both atorvastatin and ACF-2. HMG-CoA reductase, as the rate-limiting enzyme in lipid metabolism, catalyzes the conversion of HMG-CoA to mevalonic acid, leading to increased cholesterol synthesis. The elevated HMG-CoA reductase level in the model group indicated higher cholesterol synthesis, while the control group, drug group, and ACF-2 high-dose group showed significantly lower levels, suggesting a lower cholesterol synthesis rate in vivo. These comprehensive findings suggest that both the drug atorvastatin and fucoidan protect the activity of lipid metabolism enzymes, maintain higher lipid clearance rates, and achieve their anti-hyperlipidemic effects by controlling cholesterol synthesis.

Moreover, this study considered the role of intestinal flora, as it has been reported to be associated with the status of hyperlipidemia, and its relative abundance undergoes changes during therapeutic treatment in both animal experiments and clinical studies [25]. Previous research has shown that therapeutically treated mice with improved hyperlipidemic parameters exhibit a reduced ratio of Firmicutes/Bacteroidetes [26,27]. In our research, the drug group displayed a lower Firmicutes/Bacteroidetes ratio compared to the other high-fat diet groups, and AFC-2 showed a relatively decreased ratio compared to the other fucoidan groups and the model group. This suggests that both the drug group and the AFC-2 group may have derived some benefits from their gut flora composition. Additionally, previous studies have also indicated that the Lachnospiraceae family increases in mice following fecal microbial transplantation, leading to improved lipid metabolism [28,29]. In our research, an unidentified Lachnospiraceae genus showed an increased relative abundance in all high-fat diet-fed groups, except for the drug group. This suggests that the high-fat diet may promote the proliferation of the Lachnospiraceae genus to consume fat, while the administration of atorvastatin interrupted this natural response caused by the high-fat diet. Notably, the drug group displayed reduced diversity in gut flora, whereas AFC-2 had the advantage of maintaining the diversity of gut microbiota. The lower sulfated content of AFC-2 may enhance its utilization by gut microbiota compared to its higher sulfated counterpart, AFC-1.

In conclusion, our study demonstrates a dose-response relationship in the anti-hyperlipidemic effect for the fucoidan derived from Icelandic brown algae *Ascophyllum nodosum*, whether in its crude extract or two fractionated components. Among them, the high dose of ACF-2 showed a significant anti-hyperlipidemic effect in all measurements, presenting a significant difference compared to the model group (*p* < 0.05 for the final body weight and *p* < 0.01 for the rest indicators) but no significant difference compared to atorvastatin. This indicates that ACF-2’s anti-hyperlipidemic effect is comparable to atorvastatin. Fucoidan administration was found to help maintain the activity of oxidative stress-related enzymes and the enzymes engage in lipid metabolism. It was also found to refrain cholesterol synthesis-related enzyme activity, and mitigates the decline in gut microbiota diversity observed in the drug group. All of these aspects may contribute to the anti-hyperlipidemic function of fucoidan.

However, we acknowledge that our study only investigated the anti-hyperlipidemic mechanisms at the enzyme activity level due to the cycle, we did not explore in depth the signaling pathways and protein expression levels in vivo such as the Nrf2 and AMPK signaling pathways. Moreover, the simultaneous use of hyperlipidemic model mice has some limitations, with a low number of effective animals due to feeding and handling. These aspects will be the focus of our future research endeavors. Hyperliidemia is also an important risk factor for the induction of inflammatory manifestations and subsequent inflammation related chronic disorders, since a continuous and unresolved inflammatory response is usually observed in such hyperlipidemic conditions, which usually concludes to atherosclerotic and athero-thrombotic events [30]. Several algae compounds have been thoroughly studied for their anti-inflammatory potential in such pathological conditions [31], which also suggests that further studies on the anti-inflammatory and antithrombotic benefits of AN-FUC in such conditions is also of great importance and will compliment the current findings of their anti-hyperlipidemic effects, as one of the future perspectives of the present study.

## 4. Materials and Methods

### 4.1. Biological Material and Reagents

*Ascophyllum nodosum* was collected from Iceland. The crude fucoidan (AN-FUC) prepared by enzymatic hydrolysis was provided by the National Seaweed Processing Technology Research and Development Center [32]. Monosaccharide standards (L-fucose, L-arabinose, L-rhamnose, D-galactose, D-mannose, D-xylose, D-glucose, galacturonic acid, glucuronic acid) were from Sigma-Aldrich Co. LLC. (Billerica, MA, USA). Reagent kits for measuring the blood glucose level, total cholesterol (TC), triglyceride (TG), low-density lipoprotein cholesterol (LDL-C), high-density lipoprotein cholesterol (HDL-C), glutathione peroxidase (GSH-Px), super oxide dismutase (SOD), malondialdehyde (MDA), peroxidase (POD), catalase (CAT), lipoprotein lipase (LPL), hepatic lipase (HL), HMG-CoA reductase, lecithin cholesterol acyl transferase (LCAT) were purchased from the Nanjing Jianchen Bioengineering Institute (Nanjing, China). HPLC mobile phase reagents were of HPLC grade and other chemicals were of analytical grade.

### 4.2. Isolation, Purification, and Compositional Analysis of A. nodosum Fucoidan

To further fractionate the crude fucoidan from *Ascophyllum nodosum* (AN-FUC), a DEAE-Sepharose fast flow column was employed [33]. The eluent obtained from the column was used to determine the polysaccharide content using the phenol sulfuric acid method [34]. The major peaks corresponding to fucoidan were collected, and the separation process was repeated to obtain an adequate quantity of the fucoidan fraction. Subsequently, the fractionated fucoidans underwent dialysis and freeze-drying. To determine the sulfate content of each fucoidan fraction, the gelatin barium chloride method was used [35]. For the analysis of monosaccharide composition, hydrolysis of the fucoidans was performed, and the resulting PMP-labeled monosaccharides were subjected to HPLC analysis [36].

General characterization of the fucoidans was conducted using Fourier transform infrared (FT-IR) spectroscopy. Two micrograms of each sample, which had been previously dried to a constant weight, were mixed with dried potassium bromide and pressed into sheets for FT-IR scanning. A Nicolet-Nexus 470 Fourier infrared spectrometer with a scanning range from 400 to 4000 cm^−1^ was utilized for this analysis. Furthermore, 13C Nuclear Magnetic Resonance (NMR) spectroscopy was employed to analyze the fucoidan samples. A Bruker AVANCE III NMR spectrometer (Billerica, MA, USA) operating at a frequency of 500 MHz and a temperature of 25 °C was utilized for the NMR analysis [37].

### 4.3. Animal Experiments

Male Kunming mice weighing 20 ± 2 g were obtained from the Animal Experimental Center of Dalian Medical University (Dalian, China). The mice were acclimated to the laboratory environment for 7 days prior to the commencement of the experiment. The mice were housed under controlled conditions of 12-h light/dark cycle at a temperature of 25 ± 2 °C. Standard diet and water were provided to all animals throughout the acclimation period. The mice were randomly divided into twelve groups, each consisting of eight individuals. The groups were as follows: low-dose AN-FUC, AFC-1, and AFC-2 group were administered 100 mg/kg·d of AN-FUC, AFC-1, and AFC-2, respectively; medium-dose AN-FUC, AFC-1, and AFC-2 groups were administered 200 mg/kg·d of AN-FUC, AFC-1, and AFC-2, respectively; and high-dose AN-FUC, AFC-1, and AFC-2 groups were administered 400 mg/kg·d of AN-FUC, AFC-1, and AFC-2, respectively. Both the control group and the model group received saline. The drug group received atorvastatin calcium tablets through gavage at a dose of 5 mg/kg·d. Except for the control group, all other groups were fed a high-fat diet.

During the experiment, the body weight of each mouse was recorded daily at the same time. After four weeks, the mice underwent a 12-h fasting period, and blood glucose levels were measured by collecting blood samples from the lateral tail vein. Following blood collection, the mice were euthanized, and terminal blood samples were collected. The liver and gut of each mouse were aseptically removed and collected.

The collected blood samples were subjected to centrifugation at 3000 rpm for 15 min, and the resulting supernatant serum was used for the determination of total cholesterol (TC), triglycerides (TGs), high-density lipoprotein cholesterol (HDL-C), and low-density lipoprotein cholesterol (LDL-C) using commercially available kits according to the provided instructions.

The livers were rinsed with sterile saline and weighed after removing excess water using filter paper. The liver index was calculated using the following formula: liver index % = (liver weight/body weight) × 100%. Livers from five randomly selected mice from each group were partially sliced and fixed in 10% neutral formaldehyde. All the rest of the liver tissues and gut samples were preserved in liquid nitrogen before further assays and analyses. The study was approved by the Ethical Committee of Dalian Ocean University (protocol code: DLOU20191003, 12 October 2019).

### 4.4. Enzyme Activity Assay

The activity of enzymes related to oxidative stress and lipoprotein metabolism was assessed using the following methods. For the oxidative stress assay, mouse liver tissue weighing 0.5 g was mixed with 4.5 mL of saline. The mixture was homogenized in an ice water bath and subsequently centrifuged at 4000 rpm for 15 min. The resulting supernatant was collected and utilized for the measurement of malondialdehyde (MDA) levels, as well as the activities of catalase (CAT), peroxidase (POD), glutathione peroxidase (GSH-PX), and superoxide dismutase (SOD). The measurements were performed according to the instructions provided with the respective reagent kits. To determine the activities of key enzymes involved in lipoprotein metabolism, liver tissue was homogenized using the same method as mentioned above, resulting in a 10% homogenate (*w/w*). The activities of lipoprotein lipase (LPL) and liver lipase (HL) were assessed using a total lipase test kit. Additionally, the levels of lecithin cholesterol acyl transferase (LCAT) and 3-hydroxy-3-methylglutaryl coenzyme A reductase (HMG-CoA reductase) in the liver homogenate were measured using the ELISA method with specific kits. The experiments were conducted following the instructions provided by the respective reagent kits, all of which were purchased from Nanjing Jiancheng Bioengineering Institute.

### 4.5. Histological Observation of Liver Tissues

Liver tissues of five randomly selected mice from each group were fixed using 10% neutral formaldehyde solution, embedded in paraffin, sectioned, and stained with hematoxylin eosin reagent. The histological sections were observed using an inverted fluorescence microscope.

### 4.6. 16S rRNA High-Throughput Sequencing of the Gut Flora

Genomic DNA of the mice gut microbiota was extracted using the CTAB method according to the manufacturer’s protocol. The V3-V4 domain of the 16S rRNA gene was amplified using primers 341F (CCTACGGGNGGCWGCAG) and 805R (GACTACHVGGGTATCTAATCC). The PCR amplification product was then recovered and purified. Subsequently, a sequencing library was constructed. The qualified libraries were sequenced using the Ion S5™ XL sequencing platform (Thermo Fisher, Waltham, MA, USA).

### 4.7. Statistical Analysis

The data were presented as means ± standard deviation. In order to test the normal distributability of the data, statistical analysis was performed using a one-way analysis of variance (ANOVA) followed by a Duncan multiple comparison test. Statistical significance was determined at the level of *p* < 0.05 or 0.01 (SPSS Statistics 21.0, IBM, New York, NY, USA).

## Figures and Tables

**Table 1:** Compositional information of each fucoidan fractions (% of dry weight).

Component	Carbohydrate Content (%)	Sulfate Content(%)	Purity (%)	Monosaccharide Composition (%)							
				Fuc	Gal	Man	Glu	Rha	Xyl	Glu-UA	Gal-UA
AN-FUC	62.80 ± 1.21	20.48 ± 0.72	83.28	58.98	5.08	5.03	2.04	6.29	16.15	6.43	N.D
AFC-1	57.77 ± 1.44	27.84 ± 0.81	85.61	54.36	6.10	5.67	1.50	6.23	19.25	6.89	N.D
AFC-2	76.18 ± 1.02	17.60 ± 0.56	93.78	57.25	7.03	6.25	N.D	6.49	16.76	6.22	N.D

Note: Fuc: Fucose; Gal: Galactose; Man: Mannose; Glc: Glucose; Rha: Rhamnose; Xyl: Xylose; GlcA: Glucuronic acid; Gal-UA: Galacturonic acid.

**Table 2:** Effects of drug and fucoidan administration on body weight, liver index, and blood glucose level of mice. Data are expressed as the means ± SEM (*n* = 8). (**: *p* < 0.01 compared with the model group, *: *p* < 0.05 compared with the model group; ##: *p* < 0.01 compared with the control group, #: *p* < 0.05 compared with the control group; △△: *p* < 0.01 compared with the drug group, △: *p* < 0.05 compared with the drug group. L, M, H represent low, medium, high-dose group, respectively).

Group	The Initial Body Weight (g)	The Final Body Weight (g)	Liver Index (mg/g)	Blood Glucose Level (mmol/L)
Control	18.80 ± 1.06	25.49 ± 1.99 **	3.62 ± 0.16 ** △	4.48 ± 0.61 ** △
Model	19.50 ± 0.96	32.09 ± 3.23 ## △	4.55 ± 0.12 ## △△	7.92 ± 0.62 ## △△
Drug	19.26 ± 1.22	25.94 ± 1.22 **	3.97 ± 0.10 ** #	5.82 ± 0.71 ** ##
AN-FUC L	18.88 ± 2.11	28.95 ± 3.73	4.45 ± 0.16 ## △△	6.58 ± 0.86 ** ##
AN-FUC M	19.34 ± 1.26	27.52 ± 2.87	4.39 ± 0.16 * ## △△	7.58 ± 0.99 ## △△
AN-FUC H	18.74 ± 0.73	26.66 ± 2.72 *	4.35 ± 0.13 * ## △	7.08 ± 0.40 ## △
AFC-1 L	18.66 ± 2.12	26.50 ± 2.44 *	4.34 ± 0.22 * ## △	6.67 ± 0.73 * ##
AFC-1 M	20.19 ± 1.71	29.08 ± 3.56	4.33 ± 0.17 ** ## △	6.40 ± 0.91 ** ##
AFC-1 H	19.46 ± 1.01	28.81 ± 3.11	4.04 ± 0.23 ** ##	6.23 ± 0.59 ** ##
AFC-2 L	18.93 ± 1.15	27.20 ± 3.17	4.16 ± 0.22 ** ##	5.74 ± 0.33 ** ##
AFC-2 M	19.38 ± 1.34	26.55 ± 1.99 *	4.03 ± 0.12 ** ##	6.02 ± 0.73 ** ##
AFC-2 H	19.20 ± 0.90	26.77 ± 1.56 *	3.87 ± 0.29 **	5.60 ± 0.67 ** #

## Data Availability

Not applicable.
